# Three-Dimensional Outdoor Analysis of Single Synthetic Building Structures by an Unmanned Flying Agent Using Monocular Vision

**DOI:** 10.3390/s21217270

**Published:** 2021-11-01

**Authors:** Andrzej Bielecki, Piotr Śmigielski

**Affiliations:** 1 Institute of Computer Science, Faculty of Exact and Natural Sciences, Pedagogical University in Kraków, Podchorążych 2, 30-084 Kraków, Poland; 2 Humtap Inc., Sarego Street 26/16, 31-047 Kraków, Poland; piotr.smigielski@gmail.com

**Keywords:** autonomous flying agents, urban-type scene, 3D scene representation, scene analysis

## Abstract

An algorithm designed for analysis and understanding a 3D urban-type environment by an autonomous flying agent, equipped only with a monocular vision, is presented. The algorithm is hierarchical and is based on the structural representation of the analyzed scene. Firstly, the robot observes the scene from a high altitude to build a 2D representation of a single object and a graph representation of the 2D scene. The 3D representation of each object arises as a consequence of the robot’s actions, as a result of which it projects the object’s solid on different planes. The robot assigns the obtained representations to the corresponding vertex of the created graph. The algorithm was tested by using the embodied robot operating on the real scene. The tests showed that the robot equipped with the algorithm was able not only to localize the predefined object, but also to perform safe, collision-free maneuvers close to the structures in the scene.

## 1. Introduction

The problems that are studied by the authors in this paper belong mainly to the class of “search and analyze” missions designated for autonomous agents operating in 3D space. An Unmanned Aerial Vehicle (UAV) is an example of such an agent. 3D scene understanding is studied also in the context of wheeled autonomous robots. Searching for a predefined object and performing an analysis encompasses tasks like inspecting objects in urban or industrial areas [1], and also rescue missions, where autonomous agents were applied more often in recent years [2,3]. Furthermore, greater and greater demand for autonomous robots is observed in the context of the Moon and Mars exploration [4]. Navigation in an unknown environment is critical for autonomous robots in the context of executing such complex tasks. An autonomous robot designed for such missions needs to be equipped with cognitive autonomy [5]. It contains certain tools for understanding the scene as well as means of precise, collision-free navigation between obstacles [6,7].

The existing systems of the scene understanding are based on various approaches. Some of them consist in color analysis by using Markov processes [8]. Local feature descriptors determine parts of the image to retain local features, whereas global place descriptors are based on color histograms or principal component analysis [9]. Laser sensors and RGB-D cameras are used for metric calculation of the robot position [9,10]. The scene can be also understood by using neural networks that recognize model objects, such as cars, or by using semantic segmentation based on pixel categorical labeling [11].

The problem of robot navigation under the constraint of limited data from sensors was also widely studied in the literature [1,3,12]. Monocular vision can be a sufficient source of data for a certain class of tasks executed by an autonomous mobile agent. Such tasks include recognition of an area for safe landing [13,14], or robot’s orientation and navigation in the case of loss of GPS signal or inaccuracy of data received from GPS system [15] as well as wide baseline loop closing and relocalization. The ORB-SLAM monocular system, for instance, enables tracking, mapping, relocalization, and loop closing. The strategy is based on selecting the points and keyframes that leads to excellent robustness and generates a compact and trackable map that only grows if the scene content changes. This allows lifelong operation [16]. Analysis of flying robots with limited sensory equipment, in particular those equipped with monocular vision only, is especially important when Micro Aerial Vehicles or Nano Aerial Vehicles are considered because such robots impose additional significant constraints on their total mass, a significant part of which are batteries and sensors on the board.

This paper presents a solution for complex recognition and analysis tasks with the utilization of monocular vision. In the problem formulation, it is assumed that a robot searches through the terrain to locate a particular object (or group of objects), a picture of which was provided as input data. After searching is completed with success, the robot constructs a 3D model of the objects (buildings) and their spatial relations. The constructed model can be used for a more thorough investigation; among others, it allows the robot to conduct collision-free navigation close to the objects located on the scene. The conducted field tests showed the effectiveness of the proposed algorithm.

Referring in detail to the previous authors’ papers [7,17,18,19], this paper presents the complete cognitive vision module of 3D urban-type scene representation. In previous papers, 2D representation was worked out in detail, but 3D representation was worked out only in a very prototypical version. Furthermore, the 2D and 3D modules were designed and preliminarily tested separately, but only by using cameras without the robot. In this paper, the completely integrated version of the system is presented with a 3D module worked out in detail. The system had to deal with image distortion resulting from high altitude and perspective. Furthermore, the objects were detected in a natural, not artificial environment. Moreover, the integration of the vision module with the control module was implemented—collision-free flight under the recognized and “understood” structure—see the very end of Section 3. Thus, the novelties described in this publication can be summarized as follows:

1. Developing methods for navigating the robot. Positioning the robot in relation to the object to fly in close proximity. In this publication, it was presented on the example of a flight under a block having the structure of a triumphal arch.
2. Color coding of the analyzed scene using the HSV (Hue, Saturation-Value) palette. It was a necessary step to improve image segmentation and extraction of interesting objects from low-quality photos or photos taken under difficult conditions, e.g., shadows, changes in light intensity.
3. The vision system is enriched with robot camera image preprocessing.
4. The use of a Median Filter [20] with a 7-pixel grid, which results in blurring the image and removing noise.
5. Using Gamma correction [21] to darken the image while maintaining contrast. The purpose of this is to avoid overexposure resulting in a color shift in the photo.
6. The use of the Nearest Neighbor Graph algorithm, described in [7,17], implementation and integration it with the other modules, as well as testing in nonlaboratory conditions.
7. The construction of a 3D model of the scene in nonlaboratory conditions with implementation and integrating it with the other modules on a flying robot, as well as testing in nonlaboratory conditions. The 3D model construction algorithm was described in [18].

### 1.1. State of the Art

The state of the art of methods of scene analysis in the context of stereoscopic vision can be briefly summarized in the following way.

Research of stereoscopic vision methods is specifically profound in the area of autonomous vehicles operating in urban environments [22,23,24,25]. One of the fundamental problems is combining images from two cameras to identify which fragments belong to the same object. This task is crucial not only for distance assessment but also for object recognition, which is important for the calculation of distance towards surrounding agents operating around as well as their movement direction. One of the most important algorithms for combining scene models from images from stereoscopic vision is RANSAC, designed in 1981 [25,26]. This iterative method identifies pairs of points from both images, looking for the best match, calculating the transformation between the images at the same time. The method for building a 3D model of the scene, described in this paper, is based on using one camera. Stereoscopic vision is not applied and the model of a particular object on the scene is built based on splines showing the object from different angles. Such an approach has pros and cons compared to classic methods based on stereoscopic vision. Flying robots equipped with a single camera can be lighter and smaller, which allows for longer operation time and movement in smaller tighter spaces. On the other hand, collecting splines requires more movements from a robot as it needs to take images for particular spots around the object. Structural and syntactic methods play a crucial role in the development of intelligent systems for robot navigation and understanding of the surrounding area. An example of a well-defined structural method was proposed in [27]. It utilizes fuzzy sets for objects classification. A 2D model of an object is composed of primitives like lines and curves. For similarity assessment of the objects, represented with primitives, an affiliation function is utilized, aggregating values that are measuring similarity between particular composing elements of the models, to find an object in the scene that is matching with the one that was provided to the robot’s memory. The proposed method was intended to support a moving agent in localizing itself in the explored area by observing and analyzing objects encountered during the mission. A different approach was presented in [28], and it aimed to allow recognition of the objects observed by the robot from different angles. The method required training with the set of images of the objects exposed from various sides. After the training process, the robot equipped with that algorithm was able to identify that the observed object is the same one that was spotted before but now approached from a slightly different side. The set of object recognition methods proposed in this paper is based on structural analysis and aimed for robust object identification in complex environments. The structural analysis allows rotation and scale invariant recognition of the model provided to the robot as an input. The contextual approach applied on top of that is meant to support high accuracy of object recognition by analyzing not only objects’ shapes and location on the scene, but also spatial relations between them. That goal is accomplished by applying a graph model of the scene where spatial relations can be magnified or simplified based on the complexity of the analyzed scene. Hierarchical neural networks are also used for the scene understanding in which both the structures of the objects and their spatial distribution are taken into account [29]. In the cited paper the system of two neural networks—a deep one and the recurrent one—is used for the scene analysis. The deep network detects the presence of the target and its geometric attributes. The recurrent network provides information about the spatial distribution of the objects.

To sum up, in the context of autonomous robotics, there are numerous papers concerning the problem of recognition and analysis of single objects, but the number of papers concerning scene analysis is limited.

### 1.2. Problem Formulation

**Figure 1:**
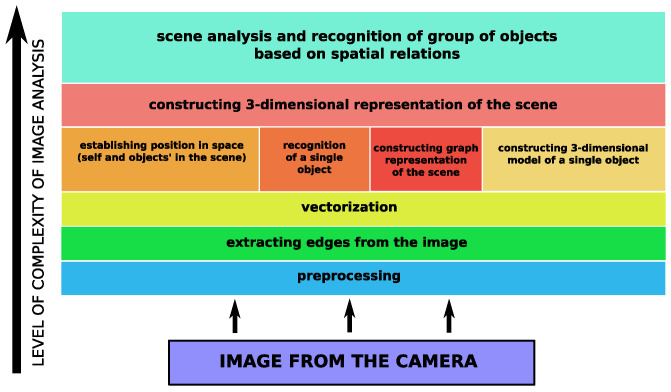
Structure of vision system of agent.

This paper discusses a complete cognitive vision module of the autonomous agent that operates in the 3D environment—for instance, unmanned flying robot—is discussed. The complete system is described (see Figure 1), and field experiments are presented.

A fully autonomous robot must, among others, understand its environment in the context of operation, primarily movement and performing planned tasks. The vision module, in addition to the physical perception of the environment through reading the image by using a camera, performs said task. This task has several stages and involves extracting interesting objects, creating their representation and modeling the spatial relationships between individual detected objects. This makes it possible to construct a 3D representation of the scene as well as its analysis and, as a consequence, understanding—see Figure 1. In this paper, a considered scene consists of only a few objects of low height. Therefore, the problem of the Earth curvature is not taken into consideration. This will be taken into account when a large scene is being analyzed and, consequently, it will be necessary to take pictures from a great height.

## 2. Materials and Methods

In this paper, the full cognitive vision module is described apart from the preprocessing problem, which is a separate topic. The extraction of an interesting object was trivial because they were colored in such a way that they differ clearly from the background—see Section 3.2. Some modules of the system were presented in earlier authors’ publications—see [7,17,18,19]. The whole system, however, was neither tested nor presented before.

The robot used for the tests has the following base characteristics. It weighs 970 g, including a 5000 mAh battery, and the diameter of the frame of the robot is 450 mm with four propellers. A single camera was installed on a stabilizing platform, which allows to switch the view between horizontal and vertical (pointing the camera forward or downward). The core components of the robot include DJI 2312E 960KV motors, DJI 430 Lite ESC (Electronic Speed Controllers) allowing peak current of 30 A, an FPV camera with 2.8 mm focal length and 120 degree view, Walkera G-2D double-axis stabilizing platform for camera, 5.8 GHz, 600 mW video transmitter, APM 2.6 controller with 433MHz telemetry module, and NEO M8N GPS module with 35 × 35 × 4 mm antenna.

### 2.1. Meta-Algorithm

The way the robot analyzes the scene is presented in the convention of the top-down approach. At the highest level of generality, the algorithm of the scene analysis and understanding can be specified as the sequence listed below.

1. Robot is provided with a preprocessed image (small map) illustrating some area to be investigated (showing one or more buildings from high altitude). 2D vector representation is calculated along with spatial relations between objects from that input image. Such data structure will be used to search for these objects in the map of overall terrain, obtained in further steps.
2. Robot flies above the terrain and takes a photo from an altitude that allows depiction of the desired area (in another implementation, robot could fly or swim lower and take a set of images that would be combined into the complete map of the area).
3. From the overall image of the terrain, the robot extracts shapes of the buildings and runs vectorization algorithm [18] to obtain more meaningful 2D representations of objects for further processing. These vectorized objects are stored in the data structure, holding information about the objects’ coordinates in the terrain. The coordinates are derived from the location of each object on the original image. As a result, the vectorized map of the area, called *vector map representation*, is obtained. The coordinates stored in the map allow the robot to navigate in the terrain to a desired location in the examined scene.
4. Robot locates the group of objects from the small map, provided at the beginning, in the *vector map representation*. To accomplish this, a syntactic algorithm of 2D object recognition is utilized [18]. As a result, using the *vector map representation*, the robot can move to the desired location where objects for investigation are situated.
5. Vehicle inspects each of the located objects taking a separate set of images for each of them. Such a set consists of one image taken from above the structure, with a camera pointing downwards (from a significantly lower altitude comparing to the moment when robot took the overall image of the area), and the rest of the images are taken horizontally aiming towards particular walls from different sides. Such images are meant to reveal the shape of the building, along with as many features (windows, holes, recesses) as possible.
6. Collected images undergoes necessary preprocessing (to reveal objects contours) and vectorization. 2D vector shapes of a building (from different sides) constitute the input for 3D vector model creation.
7. 3D models of the examined objects are used to enrich the *vector map representation*. As a result, this data structure consists of 2D vector data representing overall terrain, with coordinates of the objects observed on the scene, along with 3D vector models of particular buildings “pinned” to their locations. Such data structure is a rich source of information that allows the robot to operate in the area, performing additional tasks close to the structures without collisions.

Preprocessing of images taken by the flying robot, presented in Section 3.2, are limited to image enhancements (brightness, contrast manipulation) and extraction of particular colors. Methods of deeper image analysis and object detection, as well as algorithms of navigating a robot to a specific location, are out of the scope of this paper and will be subject to further research. At this stage of the study, the algorithm of revealing features of the walls of buildings is limited to finding holes piercing through the whole structure (i.e., pair of windows on the opposite sides of a building, located in a line of sight of a robot).

The algorithm of constructing a 3D representation of a single building requires the examined structure to be of a certain class to obtain a fully precise and comprehensive model. The structure needs to be capable of being represented as a sum, result of subtraction or multiplication of some number of basic construction elements—rectangular prisms and pyramids [19].

### 2.2. Proposed Solution

In this section, the authors present the algorithms that allow an unmanned aerial or diving robot to execute a complex task of searching and inspecting objects in the urban-like area using monocular vision only. The section begins with discussing the algorithm of vectorization (which constitutes the base for more complex algorithms described later) along with the pattern recognition method. Later, the algorithm of obtaining the 3D representation of a structure (building) is presented, followed by the description of the suggested data structure, called *vector map representation*, that keeps the information about the scene, including a 2D representation of the objects, spatial relations between them, and 3D representation of the objects that were closely inspected. These algorithms and data structures are designed to be utilized by autonomous robots operating in 3D space, either flying or diving. In the application, it is assumed that the robot is flying (Unmanned Aerial Vehicle) and the same assumption was made for the tests. The same tests could be conducted with the use of the Autonomous Underwater Vehicle. In such a case, the robot would conduct tasks related to the investigation of objects located close to, or placed upon the bottom of a lake, sea, ocean, or another reservoir.

#### 2.2.1. Prerequisites—Vectorization and Pattern Recognition

To complete the overall task, the robot has to be equipped with algorithms of vectorization and shape recognition. These methods were described in detail in [17,18]. Vectorization is used to reveal the boundaries of the structure from the preprocessed image.

Shape recognition is used to accomplish the task of searching for the given shape in the overall preprocessed map of the area. It operates on 2D vectorized pictures. Such a method has to be scale and rotation invariant to be fully usable for a robot operating on different altitudes and taking photos of objects from different directions. With the use of this method, the robot can locate the investigated object on the map of the overall terrain.

#### 2.2.2. Creating 3D Representation

**Figure 2:**
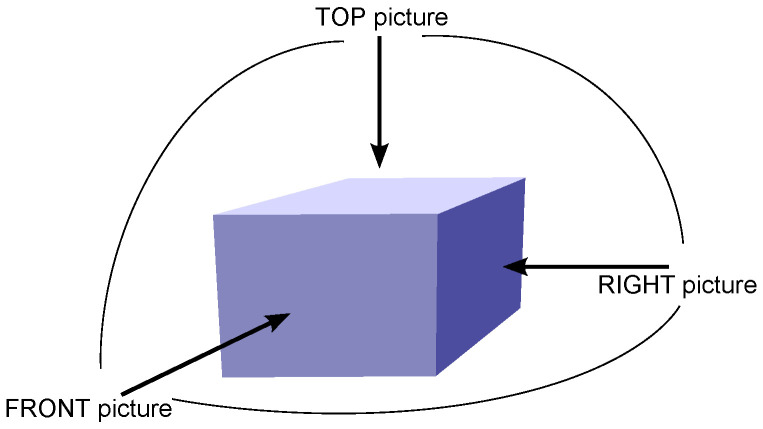
Directions from which robot takes three main images of structure.

In this subsection, the authors describe the algorithm of creating a 3D vector model of a single building that the robot finds in the area. The method is based on photographs of the structure taken by the robot from different directions. At a minimum, three pictures are required, one showing the top side of the building and two aiming horizontally towards different sides of the building, with the right angle between them (see Figure 2). Generally, the operation of the algorithm is based on the idea of composing a model of an object from projections that consist of photographed and vectorized sides of a building.

**Figure 3:**
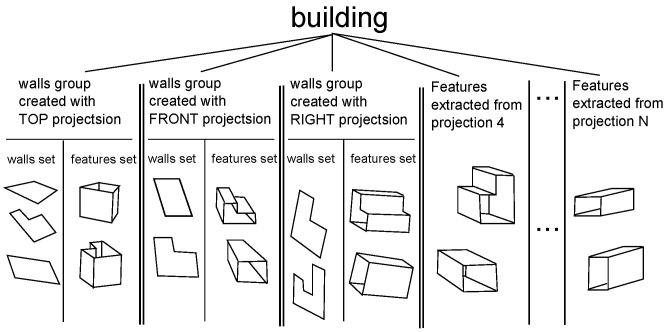
Data structure holding 3D representation of single building.

The result of this algorithm is a data structure that holds the finite set of representations of the walls of the examined building. In this structure, the walls are divided into three groups. Each group is related to one of the projections that were used for creating the model. One or more walls belong to the same group if they were obtained during the step of the algorithm in which one particular picture of the building was taken as a *reference* (see detailed description in subsection ’Creating Representation of Walls’ following in current section). It is assumed that a single wall is a flat, compact element that belongs to the outer surface of a building. Each representation of a wall is expressed by a sequence of points in 3D Euclidean space, located in the corners of the described wall. To enrich and make the data structure modeling the examined object more informative for the robot, another set of elements is added. To each of the three sets of walls, a set of representations of the features of the walls is used. Each feature (a hole piercing through the building in our application) that can be spotted by the robot’s camera aiming at a particular side of the building is itself represented by a set of walls (each of them also defined as a sequence of points in Euclidean 3D space) determining the boundaries of a hole inside building structure. There can be more sides of the building where features are discovered by the robot’s camera (besides three main sides used for 3D model creation). As a result, the data structure is also filled with the set of representations of the features that are grouped by sides of the building which they were spotted on, but not related to any of three main *reference* pictures of the sides of the building. The schema of described, composite data structure along with the idea of *wall* and *features* is shown in Figure 3.

**Figure 4:**
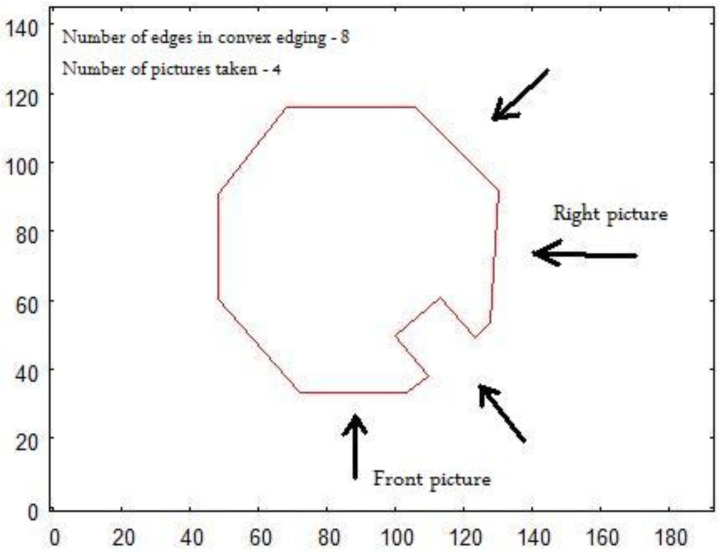
Vectorized top side of a building showing directions from which rest of pictures should be taken.

The set of vectorized pictures used as an input to the algorithm has to undergo specific restrictions:

1. All pictures, from which vector representation is obtained, have to be taken from the distance from which the overall shape of the building is revealed. Avoidance of the effect of distortion and influence of perspective is the aim of that approach.
2. One picture has to show the top side of the examined building.
3. Two of the pictures have to be taken horizontally from two sides with the angle of $90^{\circ }$ between them. One should be taken aiming towards the longest dimension revealed on the picture of the top side. The second one has to be taken aiming at the perpendicular side of the building. In the current application, the right side (looking at the front of the building) of the building is chosen.
4. To reveal the features of the walls (*holes*), the input set has to include pictures of half of the *external* side walls of the building (⌈n/2⌉, where *n* is the number of external walls). To be specific, the building has to be photographed aiming (horizontally) towards half of the sides represented by the convex hull of the top side shape (see Figure 4)

The structure of the final data set representing the final building model is reflected in the steps of the algorithm constructing the model. The method of obtaining the full model of the building is separated into the following steps:

1. Creating vector representation of walls with top side picture as a reference.
2. Creating a representation of features detected on the top side.
3. Creating vector representation of walls with front side picture as a reference.
4. Creating a representation of features detected on the front side.
5. Creating vector representation of walls with right side picture as a reference.
6. Creating a representation of features detected on the right side.
7. Creating a representation of features detected on other walls of buildingphotographed horizontally.

In the algorithm for obtaining 3D boundary representation of the structure, three vectorized pictures are taken into account—top side, front side, and the right side picture. This algorithm is conducted in three steps. In each part one building projection is taken as *reference*, and two other projections are treated as *models*. Input projections are vectorized pictures where both the shape of the building and its features are revealed.

**Figure 5:**
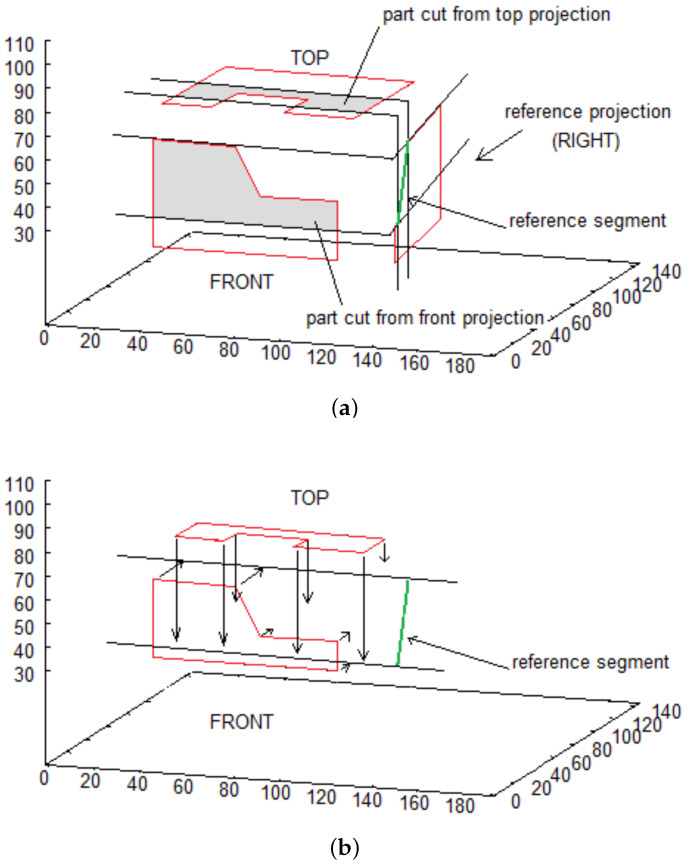

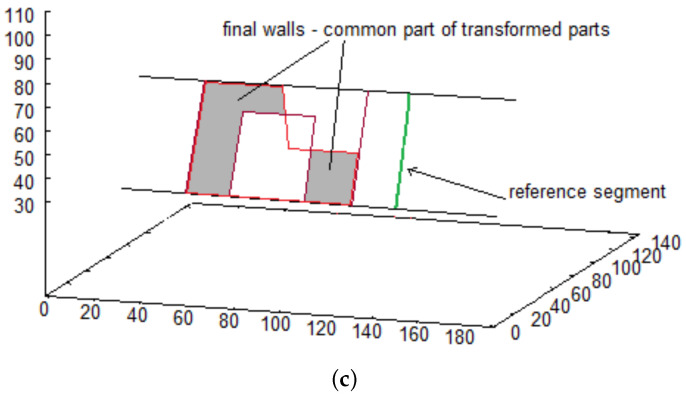
Steps of creating walls. (**a**) cutting from projections; (**b**) projecting onto a plane; (**c**) intersection of intermediate walls.

Each of the three parts of the algorithm can be described in the following steps:

1. Get two succeeding point from *reference* projection: $A=(x_{i},y_{i})$, $B=(x_{i+1},y_{i+1}),i\in \;1,\ldots ,n-1$. Such pair is called *reference segment*
2. *Reference segment* is used to cut parts from two other projections and translate obtained sequences of points as shown in Figure 5a,b,
3. Obtained walls lie on the same plane, perpendicular to reference wall (right in presented example), whose inclination is defined by the *reference segment*. Each partial wall is represented similarly to original walls—by sequence of points on its border.
4. Final step consists in calculating common part of walls (see Figure 5c)

**Figure 6:**
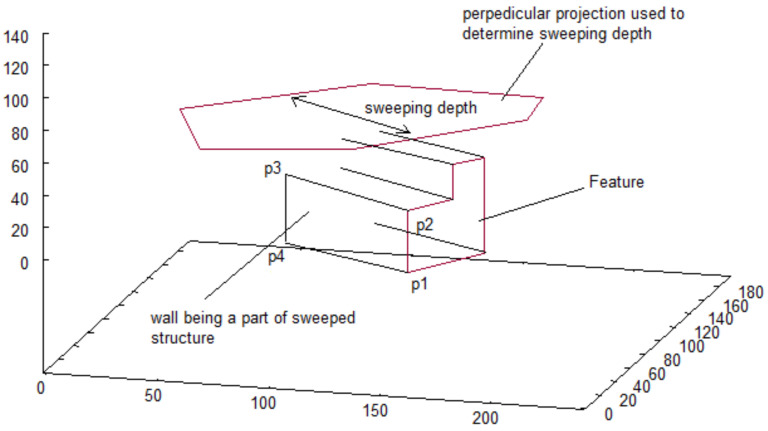
Process of feature sweeping.

For each projection on which *features* were discovered, the algorithm creates a 3D representation of each *feature*. In the input set of vectorized projections, *features* are represented in similar way, as are vectorized shapes of structure—as sequence of points in Euclidean space $(\left(x_{1},y_{1}\right),\left(x_{2},y_{2}\right),\ldots ,\left(x_{m},y_{m}\right)),$ where $\left(x_{1},y_{1}\right)=\left(x_{m},y_{m}\right)$. The difference is that the direction of points is opposite (counter-clockwise) distinguish the shape of *features* from the shape of building on projection. To obtain a 3D representation, a feature is swept along the thickness of a building. As a result, we get a set of walls that represent the inside boundaries of a hole in a building, forming a tunnel. The idea of this method is depicted in Figure 6.

### 2.3. 3D Vector Map Representation

With data that are a result of executing algorithms described above, the robot combines several pieces of vital information about the surrounding area. One is a vectorized map of overall area (*vector map representation*). The map can be informative to the robot in terms of geolocalization of separate objects in it, as well as the position of the robot in the area. The second set of data, the more complex one, consists of 3D models of chosen objects from the map. These data can be utilized by the robot to navigate close to the buildings without collisions. What is more, with information about holes in the building structure, collision-free paths across the building can be discovered. The combination of these two types of data constitutes a comprehensive data set for the robot. To combine these two data structures (2D map and 3D models of buildings) a sequence of steps is followed. Firstly, each 2D planar representation of building from the map is enriched with the third dimension coordinate with value 0, which expresses the level of the ground. Each 3D model of a building is added to the map representation and transposed so that it is located on the actual position where the building was found with pattern recognition algorithm [17,18]. The structures have to be scaled and rotated to represent the actual size and direction of placement with respect to the overall map. It can be noticed that *rotation* and *scale* values found during the recognition process can not be used here, because the recognition was conducted with a different set of pictures than the actual 3D model construction. On the basis of that observation new values of *rotation* and *scale* are calculated with the use of topside vectorized pictures of building used for 3D model construction. Having pairs of *rotation* and *scale* calculated, each vector belonging to each building model is scaled and rotated horizontally around the centroid of the model. As a result, we obtain the complex representation of the area where every building is represented by its flat shapes on the ground and some of the buildings, which were subject of deeper investigation, are represented by full 3D vector structure. The way such representation can be created is discussed in Section 3.1.

## 3. Results

The test presented in this section aims to show algorithms, described in previous sections, operating on input data similar to the one that unmanned aerial or diving robots can face. The algorithm operation and the field experiments were conducted for aerial robots.

### 3.1. Construction of the Enriched 3D Model of the Scene

**Figure 7:**
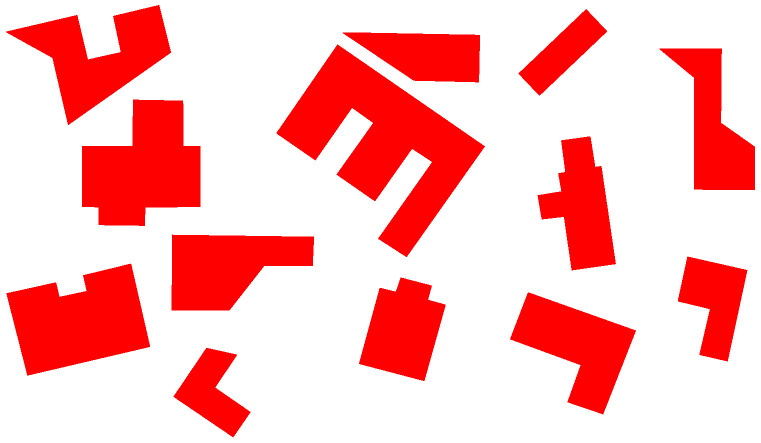
Input map for algorithm. Preprocessed picture of area to be investigated by robot.

The mentioned test scenario is described in following points:

1. Input data with the picture of building (group of buildings) consists of preprocessed bitmap showing structure (structures) from above on a white surface. This works as a small map for the algorithm
2. In place of taking a photo of the overall area from height, as it would take place when testing with the robot, another preprocessed photo is provided to the algorithm as input data. It consists of a bitmap showing a large group of objects on a white background, which represents buildings’ shapes observed from high altitude (see Figure 7).
3. Vectorization algorithm was used to obtain vector representation buildings from both pictures.
4. Recognition method was used to search objects (objects) from small pictures in the overall map.
5. When found, necessary pictures of prepared models were taken.
6. Pictures were vectorized and, with the use of result data, 3D vector models of buildings were created.
7. Data structure holding vectorized shapes of objects on the overall map is created (*vector map representation*). 3D representations of buildings are added in their respective locations to obtain the enriched model of the scene.

**Figure 8:**
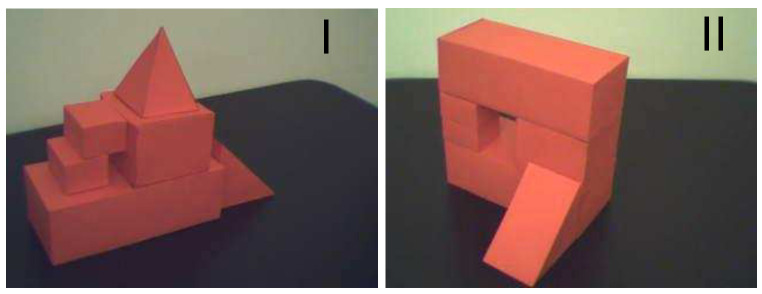
Illustrative photo of investigated structures.

**Figure 9:**
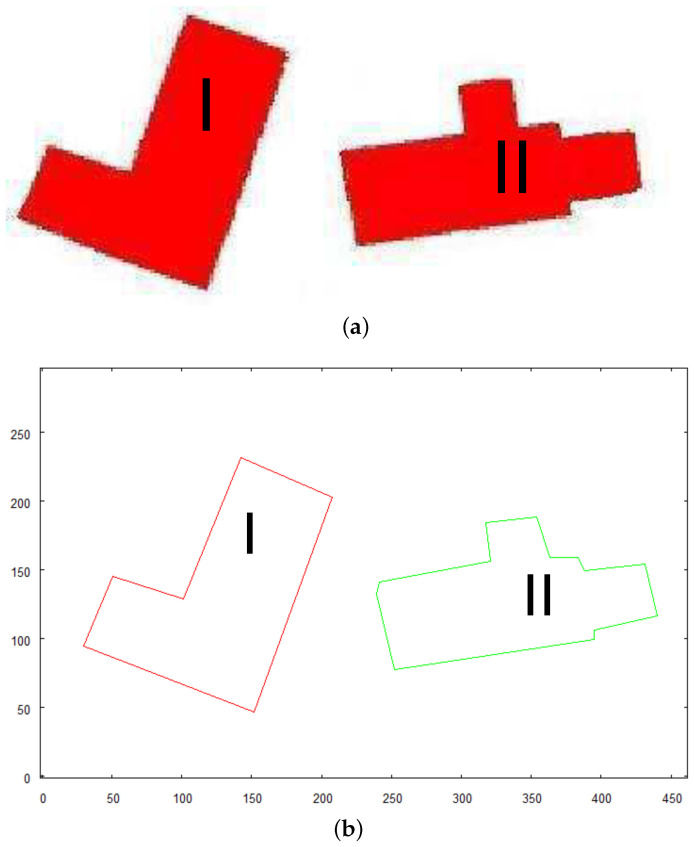
Structures that will be passed to recognition algorithm to locate them in scene. (**a**) preprocessed image; (**b**) vector representation.

**Figure 10:**
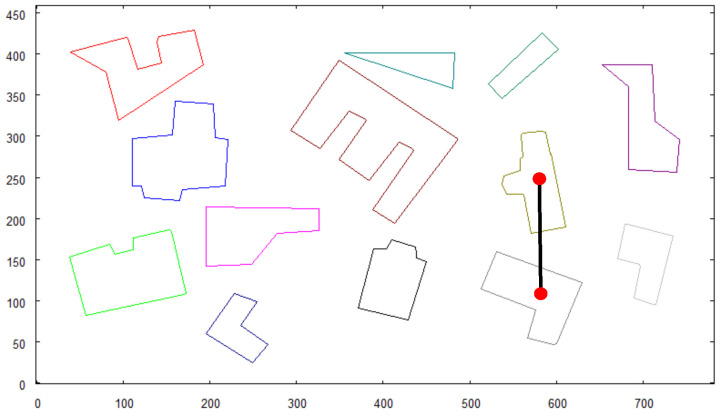
Vectorized map of terrain with objects that were recognized being marked.

**Figure 11:**
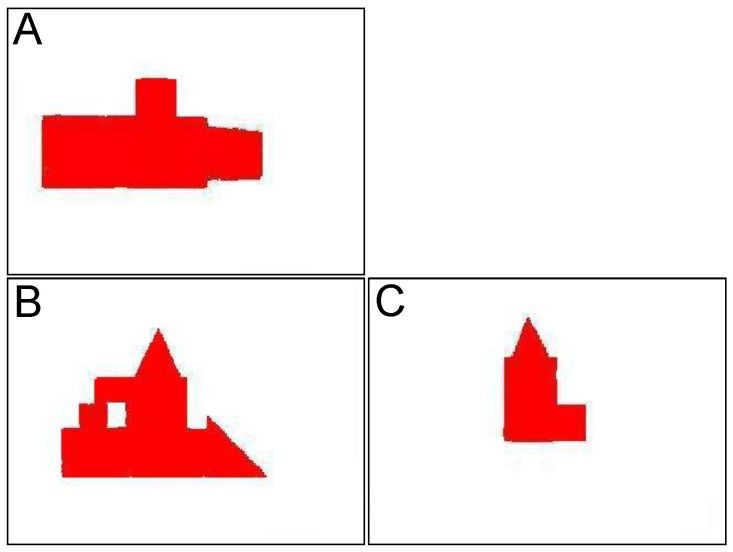
Preprocessed images of first building “I”, (**A**)—top side, (**B**)—front side, (**C**)—right side.

**Figure 12:**
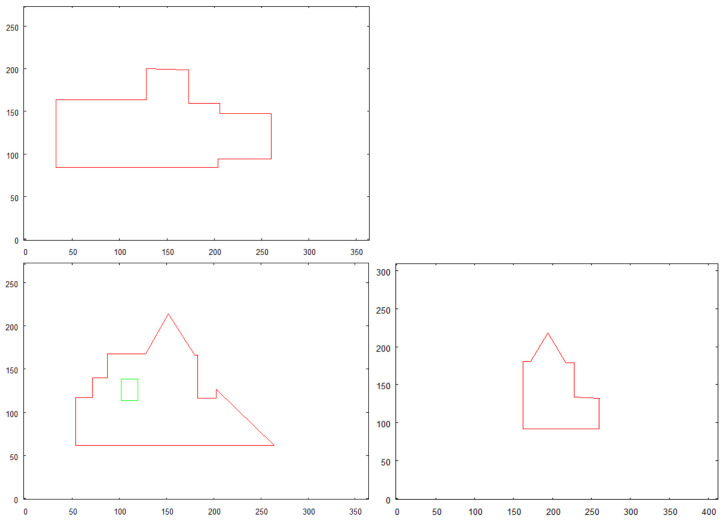
Result of vectorization of projections of building “I”.

**Figure 13:**
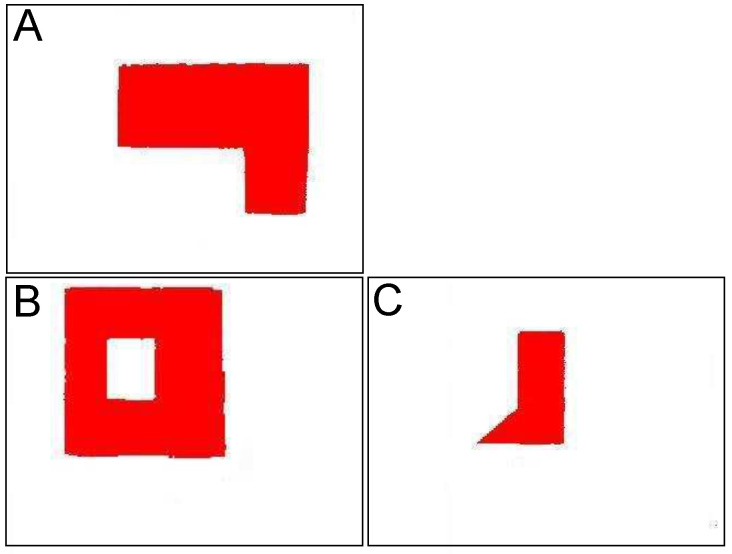
Preprocessed images of first building “II”, (**A**)—top side, (**B**)—front side, (**C**)—right side.

**Figure 14:**
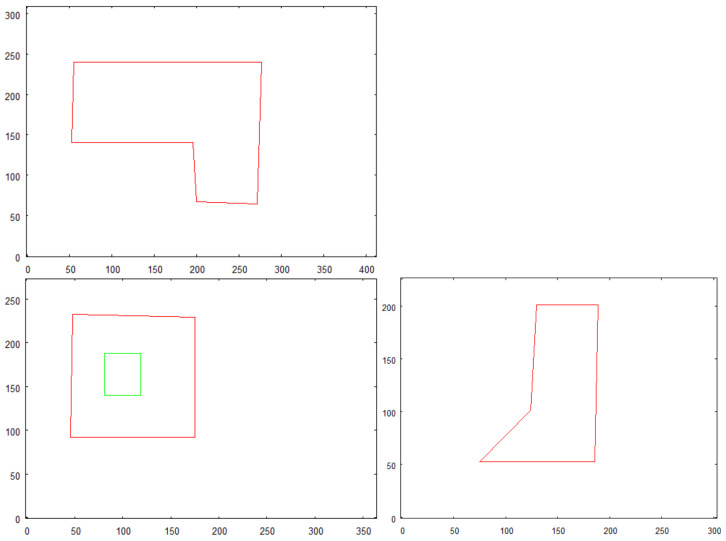
Result of vectorization of projections of building “II”.

In Figure 8, an illustrative image of structures that will be processed in this test is presented. The structures are considerably complex and each of them contains a hole, piercing through the structure, which would be identified as a *features* by 3D model building algorithm. Figure 9a shows the shapes of the same structures presented above. It can be referred to the images of these buildings being photographed by the flying robot from high altitudes. Figure 9b presents an already vectorized image that constitutes an input for recognition algorithm where these shapes will be located in the overall map of the terrain—see Figure 10. The images of the same structures, this time showing them from different angles, are shown in Figure 11, Figure 12 and Figure 13, Figure 14 respectively.

The vectorized map is presented again in Figure 10, along with markers indicating a pair of buildings that were located with the use of a pattern recognition algorithm. The spatial relationship between the objects on the predefined image (see Figure 9) is the same as in the map of the scene.

The preprocessed images of the structure “I” is shown in Figure 11. The structure is photographed while aiming towards the adjacent sides of the building—top side, front side, and right side. These images constitute an input for vectorization algorithm, whose results are shown in Figure 12.

Structure “II” goes through the same process and the results are shown in Figure 11 (preprocessed images) and Figure 12 (vectorized images).

**Figure 15:**
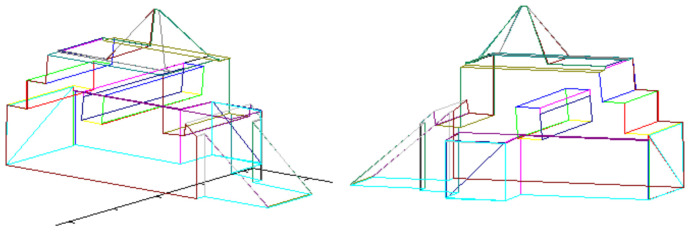
3D model of structure “I”.

**Figure 16:**
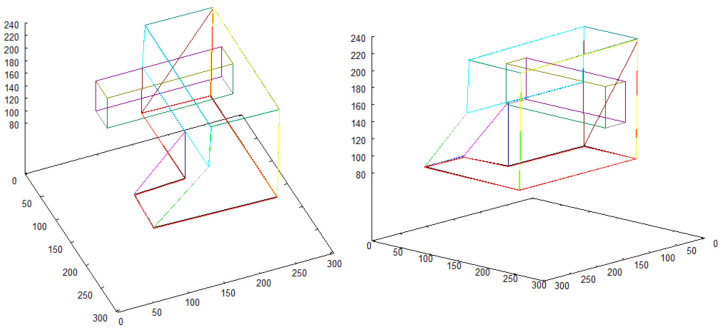
3D model of structure “II”.

Vectorized images presented in Figure 12 and Figure 14 are used for creating 3D model. The result of 3D model creation algorithm for structure “I” is shown in Figure 15. The result for structure “II” is in Figure 16.

All three 3D representations of the examined structures were added to the vector representation of the big map. The precise placement of the examined structures can be realized using data obtained during the recognition step:

1. The coordinates of recognized objects are directly related to the centroids of their vector representations on the map of the scene (see the found objects in Figure 10).
2. The scale and rotation are the direct output of the recognition method. These parameters are used to scale and rotate all vectors that create the 3D model of each structure.

**Figure 17:**
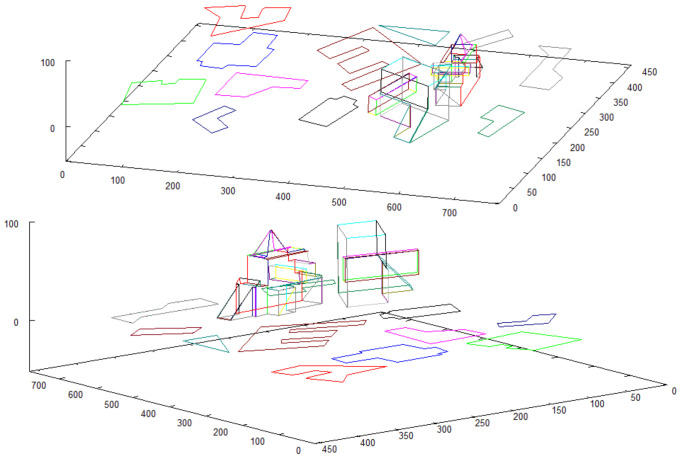
2D model of terrain, enriched with 3D models of objects that were previously found.

The result constitutes the *vector scene representation* that consists of vectorized 2D map enriched with detailed 3D models of the buildings placed in their locations on the map. This vector structure is shown in Figure 17. The model of the scene is presented from different perspectives to reveal the details and complexity of the obtained representation.

### 3.2. Algorithms Applied on UAV

**Figure 18:**
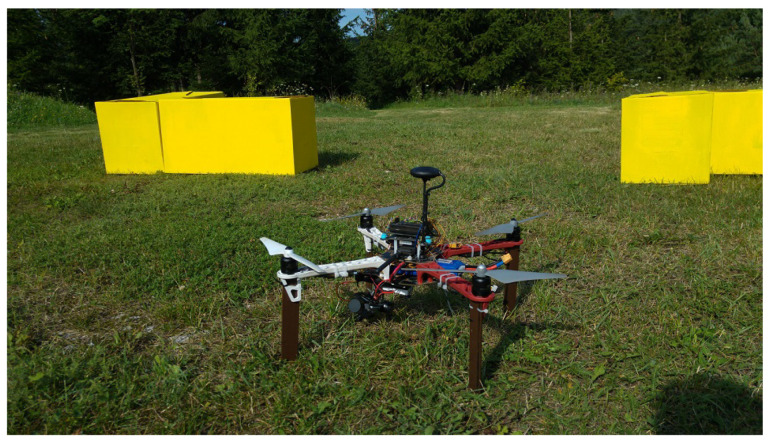
Robot used for tests.

In this section, the authors present the results of tests conducted on a UAV. The mission performed by the robot is aimed to show that the set of the algorithms discussed in this paper, facilitated by supplementary methods of scene analysis, described in this section, can allow a UAV to perform complex tasks in an urban-like environment while avoiding collisions with objects in the scene. The robot used for the tests was a lightweight quadrocopter (see Figure 18), equipped with a camera that can be pointed both forward and downwards, depending on the action taken by the robot at a particular moment in the mission. The quadrocopter was controlled by the APM 2.6 autopilot unit with an external GPS module. As it was stated in Section 2.1, the method of navigating the robot to the desired location is outside of the studies presented in this paper. In the area where GPS could not be used or the capabilities of the GPS would be limited, such as dense urban environment or even during a course of exploration of an extraterrestrial globe (i.e. the Moon), other methods of navigation could be utilized. One such method utilizes Inertial Measurement Units (IMU) based on the accelerometer to calculate whether a robot reached a target location. Combinations of IMU and visual sensors for robot positioning are also studied [30].

**Figure 19:**
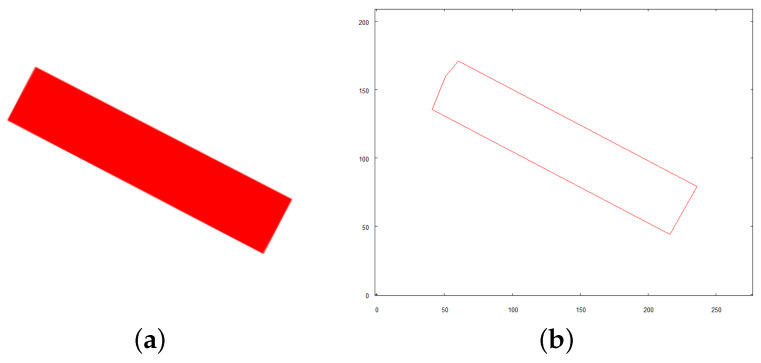
Object whose shape will be searched in scene to be identified and examined. (**a**) original image; (**b**) vector representation.

The following points highlight the essential parts of the scenario of conducted tests:

1. The robot takes off and positions itself on a predefined altitude of 9 m above the examined scene.
2. Its camera is positioned downwards and an overall picture of the scene is taken which is a base for creating the initial map of the area.
3. Objects of predefined colors (red, blue, yellow) are identified in the scene. Each of them has to be photographed directly from above to reveal its exact shape. To achieve this, the target location where the robot has to be positioned is calculated. The altitude of the target location is arbitrarily set to 4 m. Each location is expressed by distance (in meters) the robot needs to move along *x* and *y* axis of the initial map from the current position to visit first, second, and subsequent identified objects.
4. The robot visits each one of the objects hovers over it and takes an image from above.
5. Vector representations of every object are compared to the pattern to be found and closely examined (see Figure 19a for the original image of the pattern; see Figure 19b for its vector representation used for comparison).
6. Once the object is recognized, the robot enters the examination mode.
7. To examine the object that was found, the robot calculates the target positions from which it will be able to take images of the object from three crucial directions: top, front, and right side of the examined structure.
8. 3D representation of the examined object is created.
9. Robot recognizes if it is possible to fly under the examined structure starting from any of the sides that it took an image from.
10. If it is possible, the robot positions itself at a suitable position for collision-free flight under the structure.
11. Mission is complete when the robot reaches the other side of the object without colliding.

Due to the limitations of the camera installed on the robot used during the tests, the original images reveal some level of noise and saturation bias. This makes detection of object boundaries challenging and, in some cases, even impossible. To mitigate the risk of incorrect object identification the images are preprocessed in two ways:

1. Median Filter [20] is applied with the window size of 7 pixels.
2. Next, the Gamma Correction [21] is used to dim the image to avoid overexposure of some regions of the images. It is particularly necessary as the color extraction method used in the tests is based on saturation of the colors, which has to fall within specific boundaries.

Wherever the preprocessing is mentioned further in the text, utilization of Median Filter and Gamma Correction is assumed.

Following is a detailed description of each step of the test scenario.

**Figure 20:**
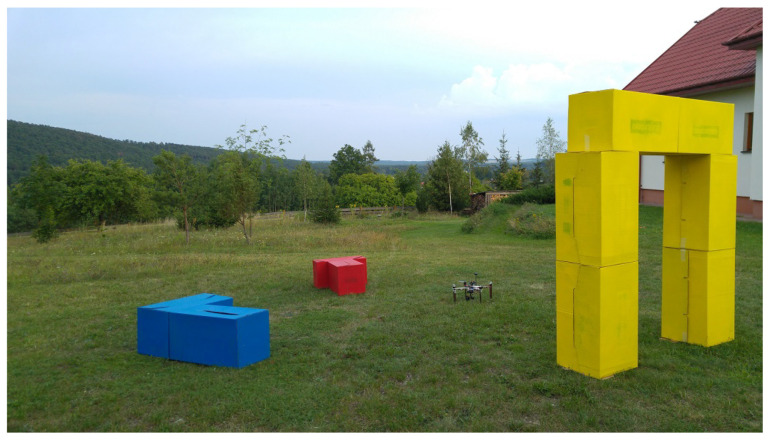
Robot ready for test execution. Alignment of objects in test area can be seen.

Figure 20 shows the robot placed in starting position in the middle of the scene.

**Figure 21:**
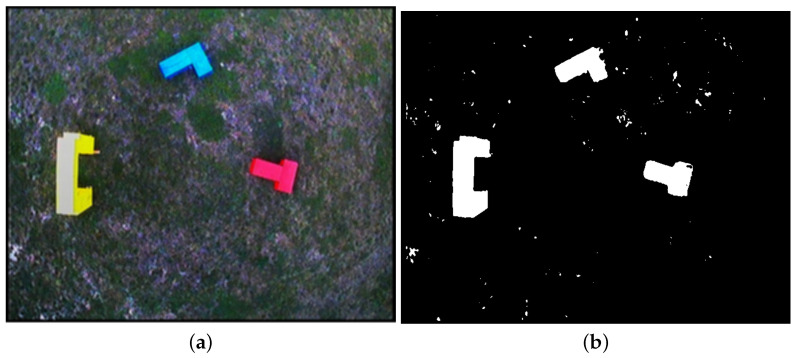
Image of scene taken from altitude of 9 m. Some unwanted artifacts on ground can be seen—see (**b**). Those will be filtered out after vector representation is created. (**a**) preprocessed image; (**b**) exposed areas where predefined colors were identified.

In Figure 21a, a preprocessed image of overall scene is shown. It was taken from the altitude of 9 m above the ground. This image, after vectorization, will constitute a base map of the scene, from which the relative positions of the objects will be derived. To identify the positions of the objects on the scene, the colors from a predefined set (red, blue, and yellow) are extracted. Figure 21b shows the regions of the preprocessed image that were identified as representing these colors.

**Figure 22:**
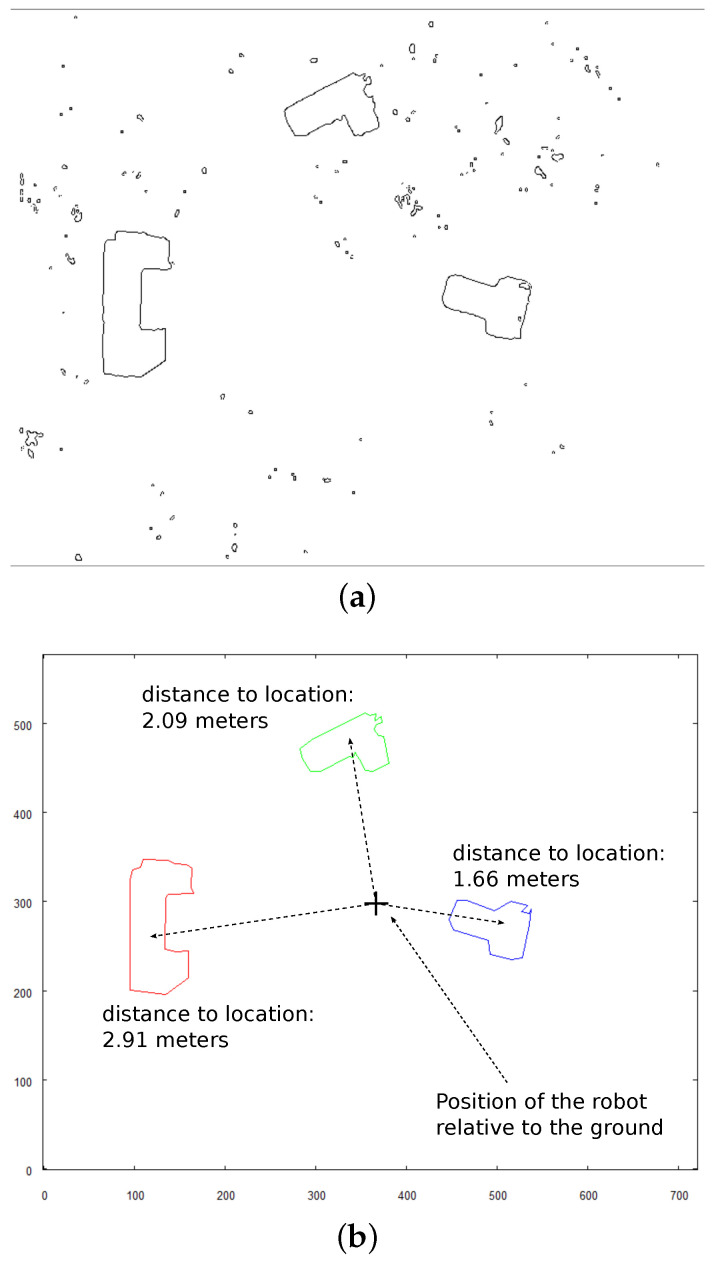
Further steps of processing of overall scene’s image. (**a**) result of applying Canny Edge Detector; (**b**) vectorized image with calculated distance to target positions for robot to take detailed images of each object.

To obtain an input for vectorization algorithm, the Canny Edge Detection algorithm [31] is applied on image from Figure 21b. The result of edge detection is shown in Figure 22a.

Figure 22b shows vector representation of the scene with the calculated horizontal distance that the robot needs to travel to position itself directly above each of the objects identified in the area. Hovering precisely over each object’s centroid will allow the robot to take the image revealing the shape of the object, which is closest as possible to its horizontal projection.

Detailed information about methods of target position estimation and robot navigation can be found in [32], section “Navigation Methods”. Although mentioned paper treats navigation in the simulated environment, the simulator was based on the same communication protocol (MavLink), and a similar controller to APM 2.6 was simulated. That allowed these algorithms to be reused in the tests on a real UAV.

**Figure 23:**
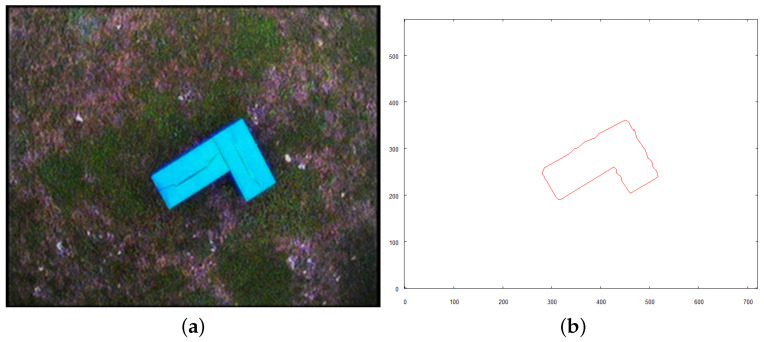
Intermediate steps to obtain vector representation of object *A*. (**a**) preprocessed image; (**b**) vector representation of object.

**Figure 24:**
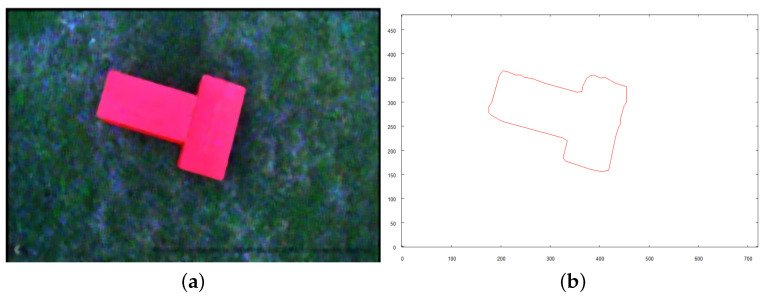
Intermediate steps to obtain vector representation of object *B*. (**a**) preprocessed image; (**b**) vector representation of object.

**Figure 25:**
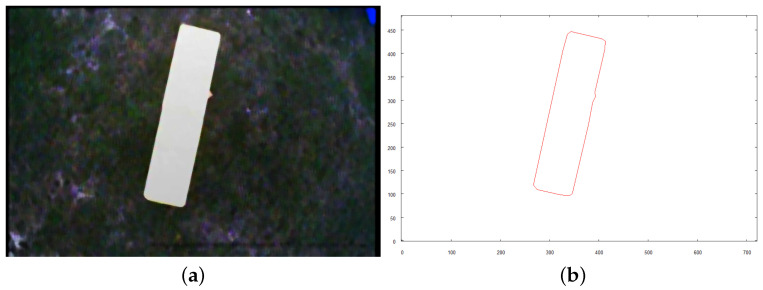
Intermediate steps to obtain vector representation of object *C*. (**a**) preprocessed image; (**b**) vector representation of object.

Each of the following figures (see Figure 23, Figure 24 and Figure 25) show preprocessed images and vectorization results for three objects found on the scene. Each source image was taken from the altitude of 4 m above the ground after the robot reached the target destination calculated based on the overall vector map of the scene (see Figure 22b).

After comparing vector representations of all three objects in the scene to the searched pattern (see Figure 19b) the match is found with object *C*. The recognition method is scale and rotation invariant. The output parameters of this method describe the scale and rotation difference between the pattern and searched object:

1. Scale: 0.606
2. Rotation: $104^{\circ }$ (clockwise)

After the searched object is identified in the scene, the robot enters the phase of 3D model building, and then tries to fly beneath the structure, if it is possible to exercise such a maneuver without collision.

**Figure 26:**
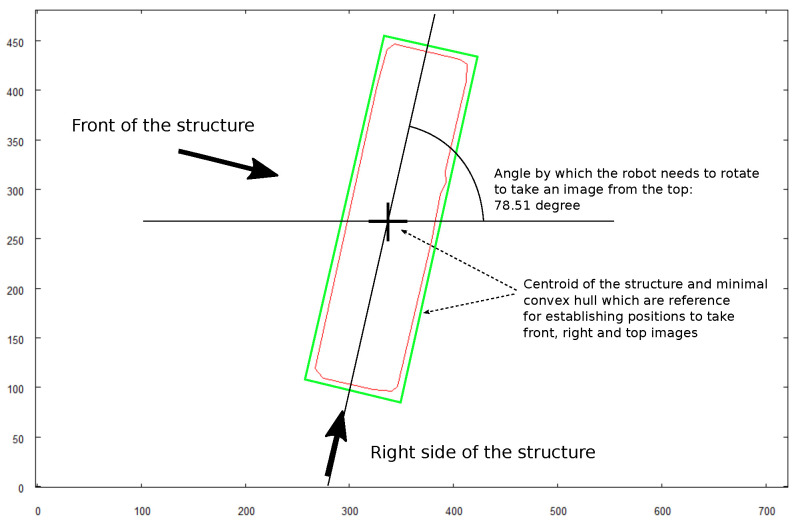
Calculating positions where robot needs to be positioned to take images for 3D model building.

Figure 26 shows the key elements that take part in calculations of the target positions for the robot from which it should take the images for creating a 3D model. All calculations are based on the image of the structure taken when the robot was hovering above and recognizing the shape of the object (see Figure 25). The initial step is establishing the minimal, rectangular convex hull of the structure. The longest side of the rectangle will be identified as the “front” of the structure. For the 3D model calculation, it is necessary to take the image of the top side when the object is positioned horizontally with respect to the front side. To achieve this, the robot needs to rotate by the calculated angle. When the robot calculates its target positions for taking “front” and “right side” images, the distance from the centroid of the object is arbitrarily set to 3 m and the altitude to 1.5 m.

**Figure 27:**
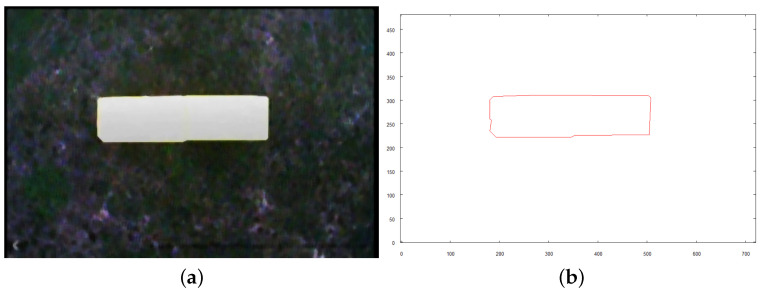
Image of TOP side of structure. (**a**) preprocessed image; (**b**) vectorized image.

**Figure 28:**
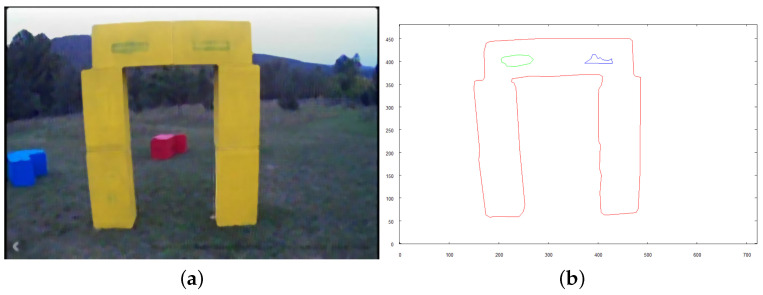
Image of FRONT side of structure. (**a**) preprocessed image; (**b**) vectorized image.

**Figure 29:**
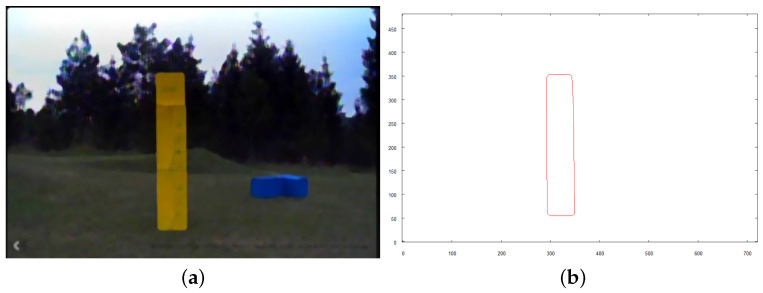
Image of RIGHT side of structure. (**a**) preprocessed image; (**b**) vectorized image.

Following figures (see Figure 27, Figure 28 and Figure 29) show the images taken by the robot after it traveled to established positions and the results of their vectorization. These vectorized images (see Figure 27b, Figure 28b and Figure 29b are used as the input for the method of creating 3D model. The extraction of the object from the image was based on the color, which was classified as “yellow”, based on the image taken for recognition (see the preprocessed image in Figure 25a). The yellow color in the image taken from above is oversaturated. To overcome this, when the robot takes an image with the camera facing downwards, very bright colors (nearly white) are included in the class of “yellow” color. On the other hand, when the camera is faced forward, such highly saturated pixels are excluded to avoid picking the sky as an element of the examined object.

**Figure 30:**
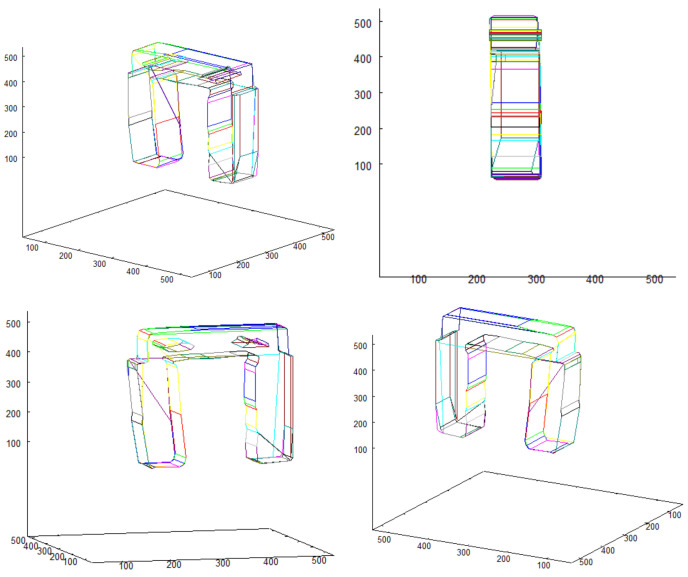
3D model of examined object presented from different angles.

Three vector representations of the images create an input for the 3D model building algorithm. The result is presented in Figure 30, in which the model is shown from different angles.

The last robot mission was to identify if any of the vertical projections of the examined structure (front or right side in the case that is described) would allow it to perform a collision-free flight under or through the structure.

**Figure 31:**
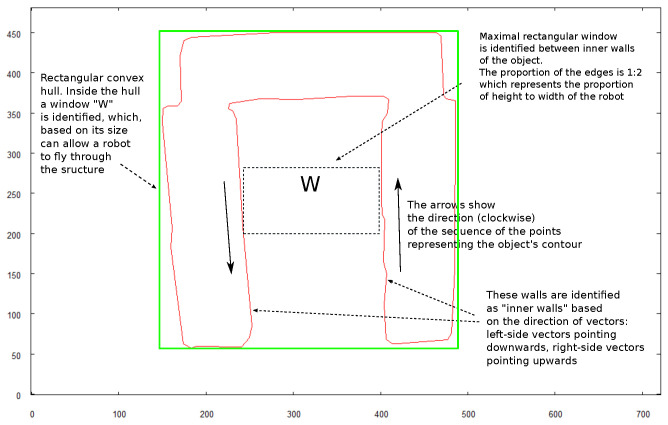
Calculating if robot can fly under structure.

The strategy for the performed test scenario was that the rectangular convex hull is calculated for each side of the object, and the robot checks if it is possible to navigate through this rectangle without colliding with the structure. The base for those calculations is the images utilized for 3D model building so it is assumed that, if the robot can fly under the structure, the starting position will be the same as the position from which the original image was taken. The core elements used for these calculations are shown in Figure 31. The size of window *W* is calculated based on the known distance of the robot from the structure (3 m) and the camera view angle.

**Figure 32:**
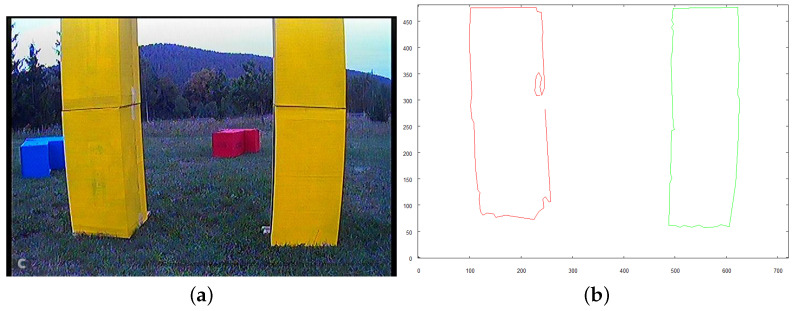
Robot approaching structure to fly underneath, 1.5 s after starting from initial position. (**a**) image from robot’s camera (**b**) vectorized image from camera.

**Figure 33:**
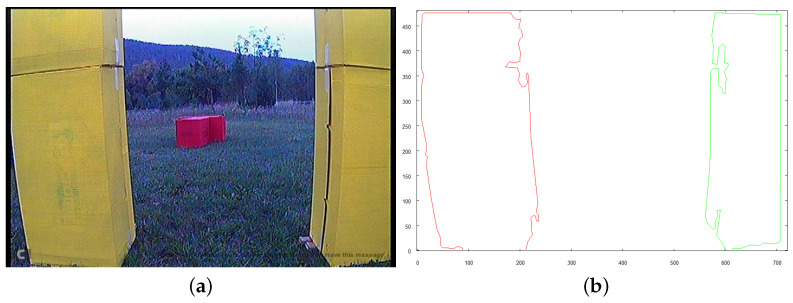
Robot approaching structure to fly underneath, 3.0s after starting from initial position. (**a**) image from robot’s camera (**b**) vectorized image from camera.

If the size of the window *W* is sufficient to allow collision-free flight through the structure, the robot initiates movement. Figure 32 and Figure 33 show images taken 1.5 s and 3.0 s, respectively, after the robot initiated the flight under the structure from the starting position.

## 4. Discussion and Concluding Remarks

We showed that a single visual sensor, capable of taking images in two directions (aimed forward or downwards), can provide enough information to the UAV to allow the execution of complex tasks. By using such a sensor, the robot is capable of understanding the spatial relations between the objects in the scene, as well as recognizing if the object of predefined shape is located in the area. Data collected using a single visual sensor can be used to enrich the model of the scene with a simplified 3D model of particular objects. Combining simple visual data with information about the robot’s position, relative to the coordinate system of the scene, can be sufficient to allow the robot to navigate between locations on the scene and perform complex missions. In the performed tests, the position of the robot was established based on input from the GPS system. However, such information could be obtained from a combination of the barometer and Inertial Measurement Unit in case GPS cannot be used, for instance, inside a building, underground, or during extraterrestrial missions. The data that are gathered during the mission, at the same time, enable the UAV to exercise collision-free maneuvers close to the object that was selected for closer examination. Commonly used approaches for scene understanding in the context of autonomous robots, shortly recalled in Introduction, are based on different techniques, and do not allow as deep a structural scene or object analysis as the approach proposed in this paper.

Further studies will include data from distance sensors (rangefinder) to collect data about the curvature of terrain photographed to obtain a more detailed map of the area. This will enable to obtain more informative data structure where spatial relations between objects will be enriched with vertical relations between them. A rangefinder will also be utilized to enrich the 3D model of the structures located in the scene, with more detailed information about the surface of their walls.

## Data Availability

Not applicable.
